# Bacterial Biosensors for Measuring Availability of Environmental Pollutants

**DOI:** 10.3390/s8074062

**Published:** 2008-07-10

**Authors:** Robin Tecon, Jan Roelof van der Meer

**Affiliations:** Department of Fundamental Microbiology, Bâtiment Biophore, Quartier UNIL-Sorge, University of Lausanne, 1015 Lausanne, Switzerland

**Keywords:** whole-cell living bioreporters, luciferase, gfp, beta-galactosidase, bioremediation, synthetic biology

## Abstract

Traditionally, pollution risk assessment is based on the measurement of a pollutant's total concentration in a sample. The toxicity of a given pollutant in the environment, however, is tightly linked to its bioavailability, which may differ significantly from the total amount. Physico-chemical and biological parameters strongly influence pollutant fate in terms of leaching, sequestration and biodegradation. Bacterial sensor-reporters, which consist of living micro-organisms genetically engineered to produce specific output in response to target chemicals, offer an interesting alternative to monitoring approaches. Bacterial sensor-reporters detect bioavailable and/or bioaccessible compound fractions in samples. Currently, a variety of environmental pollutants can be targeted by specific biosensor-reporters. Although most of such strains are still confined to the lab, several recent reports have demonstrated utility of bacterial sensing-reporting in the field, with method detection limits in the nanomolar range. This review illustrates the general design principles for bacterial sensor-reporters, presents an overview of the existing biosensor-reporter strains with emphasis on organic compound detection. A specific focus throughout is on the concepts of bioavailability and bioaccessibility, and how bacteria-based sensing-reporting systems can help to improve our basic understanding of the different processes at work.

## Introduction

Sensing techniques form an integrated part of our modern life. We like to be accurately and constantly informed about the quality, security and composition of products that we consume or encounter in our daily life. Medical tests need to provide instantaneous answers on health parameters, blood values or presence of potential pathogenic organisms. Industrial processes rely on constant physical and chemical sensing of process parameters, system inflow or outflow. Sensors come in thousand and more forms and shapes, principles and output. Future demand calls for further miniaturization, continuous sensing, rapidity, increased sensitivity or flexibility.

One of the emerging domains in sensing technology is the use of living (microbial) cells or organisms. Whereas this principle is arguable very old (for example, mine canaries were used in Roman times to sense carbon monoxide), it is only since the last twenty years that living cell-based sensing assays have gained impetus and developed into a scientific and technological area by itself. The question we would like to discuss here is why one would use living cells and organisms for sensing? What are the specific purposes for basing sensing methods on living cells and what are the advantages that cellular-based sensing can have over other sensing techniques? In this overview we will concentrate specifically on bacteria- (microbe-) based sensor (MBS) methods. We will shortly rehearse the major design principles in MBS and give some examples of potentially useful applications that have been achieved up to now. Furthermore, we will focus our attention on the concepts *bioavailability* and *bioaccessibility*, which are useful to explain the central conceptual differences between sensing based on living cells and other sensing methods.

## Microbe-based sensors (MBS)

Initiated almost twenty years ago [1], the engineering of microbial cells with the purpose of chemical detection has enormously expanded since [2-4]. The major driving force for this development has been the advance in genetic engineering techniques; the relative ease to redesign (certain) hardware components in microbial cells and to assemble synthetic genetic circuitry for sensing and producing robust output signals. Although in principle any constituent, product or reaction of living cells can form the basis for a ‘sensing device’, most research has concentrated on non-cognate so-called reporter proteins that are to be produced by the cell after specific contact or interaction with a target analyte or condition [5,6]. The use of non-cognate proteins as reporters ensures a low background in the absence of the trigger, and, ideally, a highly specific output signal [3,7,8]. In addition, the conditional synthesis of the reporter protein is an important prerequisite for a high signal-to-noise ratio.

The choice of a suitable reporter protein is dependent on the targeted application form. For example, MBS used for *in-situ* single-cell measurements often apply autofluorescent proteins as reporters [9,10]. A large variety of autofluorescent proteins with different spectral properties, maturation kinetics, photobleaching or temperature stability is now available, mostly but not exclusively based on mutants of green fluorescent protein (GFP) or DsRed [11,12]. Recently, a new type of fluorescent protein based on the YtvA protein of *Bacillus subtilis* and *Pseudomonas putida* was developed that can produce fluorescence even in the absence of oxygen, a characteristic which GFP does not have [13]. Bulk measurements of MBS have been carried out with several different types of reporter proteins [3], of which bacterial and eukaryotic luciferases have been particularly popular [7,14]. Mostly because of their relatively high quantum yields, luciferases have been the optimal choice for highly sensitive applications. Different spectral variants have been developed by mutagenesis strategies [15,16]. On the other hand, eukaryotic luciferases require substrate addition and cell membrane permeabilization in bacteria, which somewhat limits their practicality for MBS assay configurations. Bacterial luciferases have been the most applied reporters in MBS. Two different configurations have been used, one (LuxCDABE), in which the cells synthesize the substrate for the luciferase, and another (LuxAB), in which external substrate addition is needed [7,14]. Although external substrate addition is somewhat more cumbersome, it avoids false-positive stimulation of luciferase activity by membrane regeneration [17] and is less energy demanding for the cell. Other reporter proteins can be used for colorimetric or electrochemical detection [3]. Of these, beta-galactosidase is currently probably the most versatile, because a large variety of substrates is available for different detection purposes.

**Figure 1.**
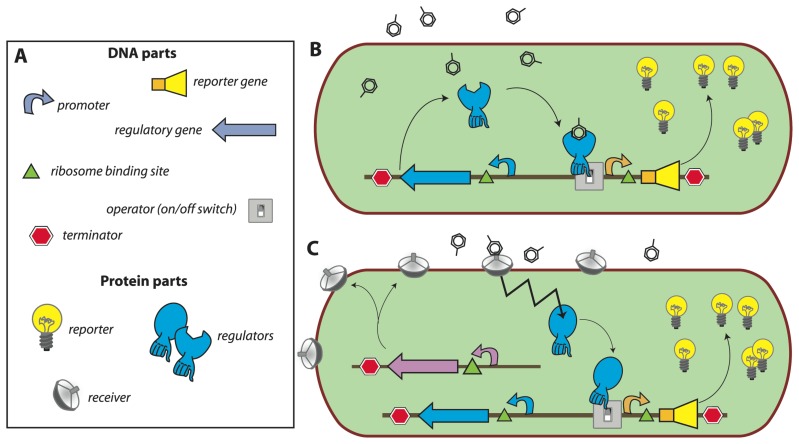
Concept of a bacterial sensor-reporter cell. **(a)** DNA parts necessary for constructing an inducible sensor-reporter circuit. Parts can be combined and assembled by genetic engineering techniques. Regulatory and reporter genes are necessary for the sensing function and system output, respectively. Promoter, operator(s), terminators, ribosome binding sites, etc. are DNA sequences needed for control of the gene expression. **(b)** Set-up in which the sensor function is provided by a single regulatory protein. In this example, the regulator protein binds the target compound and induces the transcription of the reporter gene, leading to the production of reporter proteins (signal amplification). **(c)** Set-up for separated sensor and regulator functions. In this configuration, the target compound is sensed by a periplasmic receiver protein that transmits the detection event via a signalling (e.g. phosphorylation) cascade to the regulatory protein (zigzag arrow). The activated regulator then induces reporter gene expression as before.

**Figure 2.**
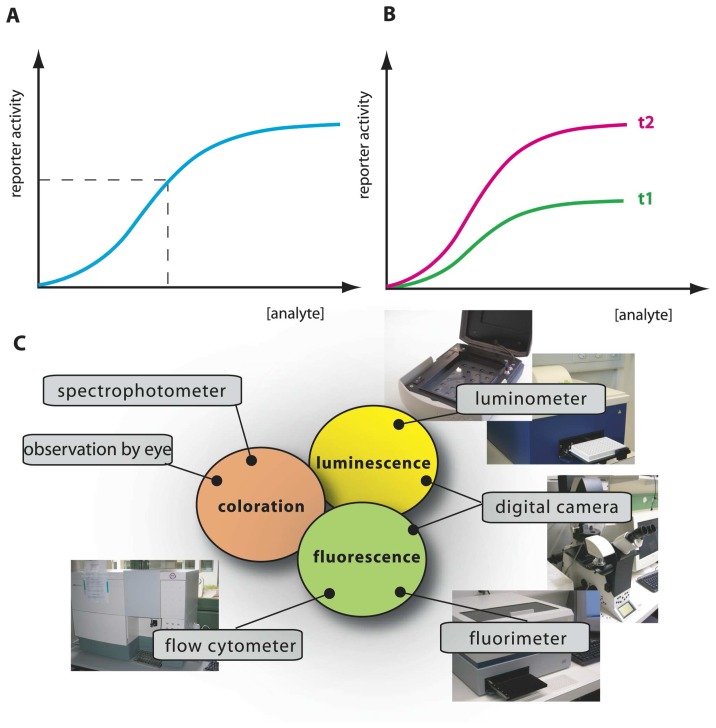
Schematic analysis of an MBS-assay. **(a)** Typical calibration curve with reporter output as a function of analyte concentration, produced from incubations with a set of known analyte concentrations. Output from an unknown sample is interpolated on the calibration curve (dotted lines), analyzed at the same time and under the same conditions, to derive a value of ‘equivalent target compound concentration’. Additional spiking assays can be performed (i.e., adding known target amounts to unknown samples) to correct for possible sample interferences or presence of toxic compounds. **(b)** Time-dependent signal calibration. MBS-assays are usually carried out in such a manner that output values are relative: dependent on incubation time and amount of cells in the assay. Her as an example curves t2 and t1 for longer and shorter incubations, respectively. For this reason, simultaneous calibration curves must accompany analysis of unknowns. **(c)** Various instruments for measuring reporter output, here shown as an example for three currently used reporter activities: fluorescence, bio- or chemiluminescence, and colorimetry.

In most of the current designs, the *de novo* synthesis of reporter protein is under control of a transcription factor, which directs the repression or induction of reporter gene expression from a dedicated site on the DNA (e.g., promoter). The sensory function can be provided by the transcription factor itself via, for instance, an internal effector binding domain that transmits target perception to forming productive interactions with RNA polymerase [7], or via a sensory protein, which subsequently transmits the perception event via a signalling cascade (e.g., phosphorylation) to the ultimate transcription regulator [18] (Fig. 1). Sensing events are thus translated and amplified in the form of reporter protein synthesis, the activity of which is generally measured in the assay (resulting in further signal amplification). The specificity of target detection is determined by the recognition specificity of the primary sensor protein or transcription factor, and by any other condition influencing the signaling cascade or acting on the same promoter [19]. The construction of the genetic circuitry for the sensor-reporter conditional switch is accomplished by established recombinant DNA technology or, more and more, by direct DNA synthesis. Dedicated resources have become available that list available biological parts and their specifications needed for the circuitry, much like catalogues of electronic parts (http://partsregistry.org/Main_Page, Fig. 1). Due to the ease of manipulation, bacteria such as *Escherichia coli* are very often used as host cells for the sensor-reporter constructs, but likewise have yeast [20] or human cell lines [21] been employed. Many different instruments can be used for the measurement of the reporter signal, and both populations of sensor-reporter cells (i.e., bulk measurements) or individual cells can serve as basis for reporter analysis (Fig. 2).

## Bioavailability

Are there specific advantages for exploiting living cells for sensory purposes rather than e.g., physico-chemical detectors, or even purified proteins and antibodies? Obviously, in order for the sensor-reporter construct to operate, the MBS need to be maintained alive and in some sort of active state and optimal environment to produce the required response. This requirement in practise puts serious constraints on the shelf-life of MBS. On the other hand, MBS are self-propagating entities and therefore relatively easy and cheap to produce. The fact that different MBS can be engineered, which solely differ in target recognition but otherwise have the same reporter output signal, may pave the way for sensing arrays while maintaining relatively simple detectors and devices [4] (box 1). The main important advantage for using MBS, however, that (for the time being) only cells themselves can provide is the integration of biological processes relevant to the target one would like to address. Cellular toxicity, for instance, is conceptually most easily determined by the cell in question itself, if we succeed in interrogating the appropriate biochemical elements in the cell. Bacterial pollutant degradation activity (another domain where MBS are used) is most accurately measured by the bacterial cells themselves, which we can translate into a useful reporter signal when directing the dedicated genetic sensor-reporter circuit to the appropriate key elements in the cell. In the following, we will thus argue that the key advance made by MBS is to analyze biologically relevant processes while providing at the same time a certain analogue (the bioavailability or bioaccessibility fraction) to classical chemically derived compound concentrations (or chemical ‘activities’). This is most easily explained in the form of the example of pollutant remediation and environmental risk assessment.

## Bioremediation and risk assessment

Environmental risk assessment is an essential tool in the investigation of polluted sites and the subsequent decision making process on the eventuality of active site remediation. In Switzerland alone, some 50′000 polluted sites have been entered in inventory – among which 4′000 may represent a danger for environment and will have to be treated in the next 15 years [22]. Obviously, there is insufficient public funding available for an extensive treatment of every site, and thus priorities have to be set on the basis of pollution exposure and risks. Current regulations most often base on total pollutant concentrations at a site for predicting risks. However, most likely only a fraction of the total amount of hazardous substance will actually have an impact on living organisms (by definition, the fraction which is available or accessible to the organisms). Therefore, the use of the total amount is likely to overestimate the risk [23]. The discrepancy between the total and the bioavailable or bioaccessible fractions is particularly significant in the case of contaminants with poor aqueous solubility (e.g., PCBs, PAHs) or very low dissociation constants (e.g., certain heavy metal precipitates). Nowadays, increasing attention is thus given to *bioavailability* assays that better predict the real exposure of specific organisms to pollutants [24].

Although the term *bioavailability* is frequently used in scientific papers, it does not always have the same definition. For this reason, other authors preferred to speak of *bioavailability processes*, to reflect the fact that various biological, chemical or physical steps influence the final outcome [24]. In this review, we will use Semple's definition of bioavailability as the fraction of a chemical in a system “which is freely available to cross an organisms's (cellular) membrane from the medium the organism inhabits at a given point in time” [25,26]. The authors further suggested using the term *bioaccessibility* to dinstinguish the fraction that could *potentially* cross the cellular membrane if the organism had access to it. A bioaccessible fraction can become bioavailable over time or in space if physical barriers that restrict access to the organism are relieved. Organisms themselves can influence the bioaccessible fraction by changing the compound mass-transfer rate to the cells [27]. For example, a bacterium metabolizing a poorly water-soluble carbon compound will deplete this from solution, which can drive further dissolution from a solid phase. Semple *et al.* argued that it would be useful to differentiate chemically active compound (bioavailable) from chemically inactive but potentially exploitable (bioaccessible), and that for risk assessment the bioaccessible fraction would be the more relevant determinant. Bioaccessibility is inherently organism-dependent [24], but its actual (numeric) value may be the same among various organisms. Therefore, model organisms such as MBSs may be useful to assay bioaccessibility.

## Bioavailability and bioaccessibility assays with MBS

**Figure 3.**
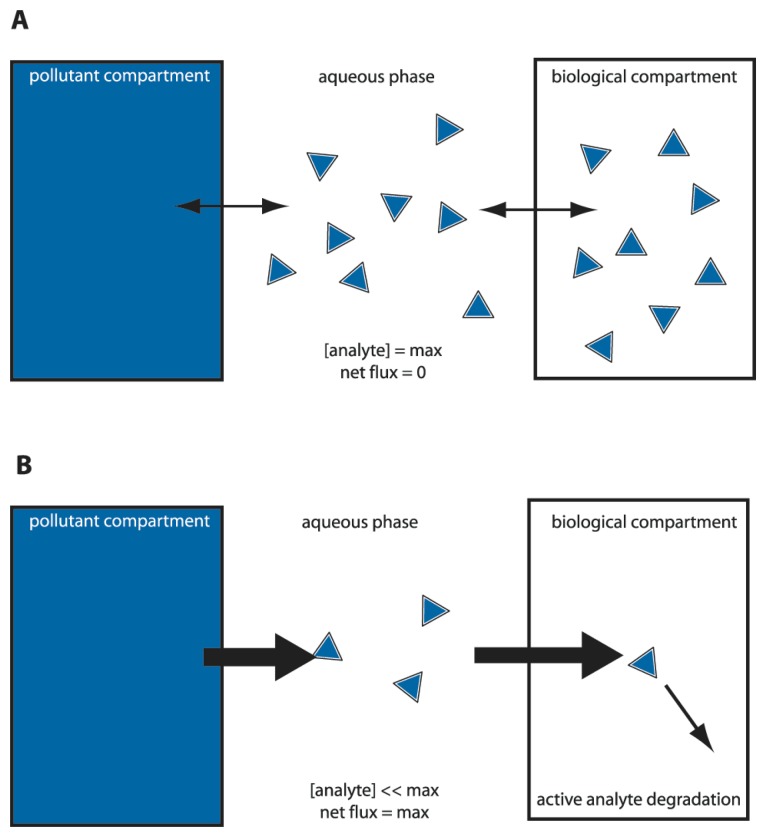
‘Equilibrium’ versus ‘sink’ sensor-reporter cells to differentiate between bioavailable and bioaccessible fractions. **(a)** Microbe-based sensors (MBS) which do not degrade the analyte rely on the aqueous phase concentration or chemical activity. An equilibrium will arise between bulk aqueous phase concentration, lipid fraction and intracellular compound concentration (the latter more or less equalling the aqueous phase concentration). The MBS can only sense the immediate or bioavailable fraction. **(b)** MBS that can degrade the analyte. By degrading the analyte, a flux is created from the pollutant compartment to the biological compartment. The MBS thus acts as a ‘sink’ and can detect part of the bioaccessible fraction. Thickness of the arrow points to the pollutant flux from one compartment to the other. The MBS cell here is depicted as a square box.

We could thus envision different types of bioassays targeting compound bioavailability and bioaccessibility. A typical MBS assay consists of incubating the cells in an aqueous sample for a particular pre-defined reaction period, after which the reporter signal is determined (Fig. 2). Because in this case the sensor-reporter cells can be assumed not to have been limited by the access of the compound in solution (i.e., no mass transfer limitation existed), they must have detected the fraction which was bioavailable to them during the assay period. We will see that this is essentially the case, although metabolic decisions in cells can still influence the behaviour of the sensor-reporter [19,28]. Bioaccessibility assays are trickier to perform, because in essence they have to somehow overcome the time or spatial barrier that prevents further compound transfer to the cells. Chemically, bioaccessibility can be tested by using so called non-exhaustive extraction techniques (NEETs). NEETs employ, for instance, Tenax or cyclodextrins to rapidly retrieve a compound fraction from the sample that is similar to the fraction metabolized by (micro-) organisms during a much longer incubation period [29,30]. For example, Dick *et al.* added [^14^C]-labeled phenanthrene or pyrene to soils, and showed that the total fraction of PAHs metabolized by bacteria in the soil during thirty days as derived from [^14^C]-CO_2_ evolution was almost the same as the PAH-amount extracted by hydroxypropyl-beta-cyclodextrin [30]. In a MBS assay, this might be imitated by using sensor-reporter cells which not only detect, but also metabolize the target compound. These cells will create a mass transfer flux during the assay and may thus more faithfully detect the bioaccessible fraction (Fig. 3). For the remainder we will discuss a number of MBS assays specifically in the light of bioavailability – bioaccessibility detection of organic chemicals.

## MBS detection of benzene, toluene, ethylbenzene and xylenes

In a number of MBS-assays so-called BTEX compounds were addressed. BTEX stands for benzene, toluene, ethylbenzene and xylene; four volatile aromatic compounds that are found in crude oil, gasoline and natural gas. BTEX are also massively produced by industry as solvent and starting materials for chemical synthesis, and are considered as one of the major environmental pollutant classes [31-33]. The four compounds have various toxic effects, including blood disorder, impact on the central nervous, reproductive and respiratory systems, whereas benzene is also a known carcinogen [34]. Because BTEX compounds are rather water soluble (e.g., up to 1.8 g/L for benzene [35]), they represent a risk for drinking water pollution [34]. On the other hand, their volatility and hydrophobicity make it hard to predict their bioavailability and bioaccessibility.

The first MBSs for the detection of BTEX and related compounds were created more than ten years ago using the regulatory protein XylR and the P*_u_* promoter from the xylene degradation pathway on the TOL plasmid of the bacterium *Pseudomonas putida* mt-2 as a conditional switch [36,37]. One of these consisted of an *Escherichia coli* strain carrying the plasmid pGLUTR, which expresses firefly luciferase (*luc* gene) from the XylR-P*_u_* system [38]. Other MBSs for BTEX used the TodST sensor-regulatory proteins and the P*_todX_* promoter from the toluene degradation pathway of *Pseudomonas putida* F1, coupled to expression of bacterial luciferase [39-41]. Also the regulatory protein TbuT and the P*_tbuA1_* promoter from the toluene degradation pathway in *Ralstonia pickettii* PKO1 have been used as a basis for a BTEX-MBS, this time exploiting *Pseudomonas fluorescens* A506 (pTS) as a host strain expressing the green fluorescent protein (gfp) as reporter [42]. Both *E. coli* DH5alpha (pGLUTR) and *P. fluorescens* A506 (pTS) were not able to degrade BTEX compounds, whereas the MBSs employing the TodST-P_todX_ constructions was. Interestingly, the presence of other carbon substrates diminished the reporter output from *P. putida* F1-P_todX_-*luxAB* [41]. The authors explained this behaviour by assuming that multiple usable carbon substrates diluted the metabolic flux through the toluene pathway [41]. Although this can be considered as a hindrance for successful use of the MBS for bioaccessibility measurements, the system does present a faithful reaction of the cells. This implies that in this case toluene bioaccessibility is diminished because of simultaneous presence of other compounds. Even the non-degrading MBS for BTEX did not in all cases respond to the available fraction in aqueous solution, because of metabolic interference at the P_u_-promoter. This promoter is especially prone to secondary control, such as via the phenomenon of ‘exponential phase silencing’ [43]. The result of this interference is that the promoter is not induced even though sufficient toluene is present for the cell.

As outlined above, in most assays the MBS were calibrated in aqueous solution with known BTEX concentrations. The reporter signal produced from unknown aqueous sample incubations is interpolated on the calibration curve, from which a so-called BTEX-equivalent concentration can be derived (box 1). In order to appropriately estimate BTEX availability and accessibility in contaminated soils, samples have been extracted and the extract incubated in the MBS assay. Willardson *et al.* attempted to extract soils with ethanol and add the ethanol extract in the MBS-assay. A dilution of almost twenty times had to be used, at which ethanol concentration still ≈ 40% inhibition of the cells occurred. This resulted in a BTEX detection limit of 30 mg/L [38]. Other groups used soil-water extracts [44,45], and showed that toluene-equivalent concentrations determined in the MBS-assay were similar as the total concentration of ethylbenzene plus benzene in the soil-pore aqueous phase by GC-MS [45]. Dawson further compared BTEX degradation in soil over a 30-days time period and measured toluene-equivalent concentrations in the soil-water extract by their BTEX-biosensor. They showed that the MBS detected less-and-less over time as biodegradation proceeded, but no correlation was made to the total BTEX load in the soil determined by methanol extraction and GC [44]. From these studies we can thus conclude that MBS detect bioavailable fractions in soil-water extracts which are similar as the dissolved chemical concentration (except in the case of metabolic interference as discussed above). Organic extractions on the other hand, retrieve higher BTEX fractions from soil, and, therefore, MBS-assays on the organic solvent extracts provide an idea about the bioaccessible fraction. Disadvantage of use of organic phases is that they easily inhibit the cells in the assay. For this reason, the extracts have to be used in highly diluted form.

Very few studies actually investigated BTEX availability and accessibility fractions in soil without the introduction of an extraction step. In principle, an incubation of MBS cells with the sample and subsequent retrieval and measurement of the MBS reporter signal at different incubation time periods would show the immediate response (i.e., bioavailable fraction) and the slow released fraction (bioaccessible). An excellent example of this principle was provided by Leveau *et al.* [46], who analyzed fructose bioaccessibility on plant leaves. Casavant and colleagues [47] developed a similar idea for monitoring toluene availability *in planta*. However, their sensor-reporter system did not show a dosage effect, but only produced a yes-or-no signal. From the number of individual MBS cells expressing GFP isolated from the exposed plant root they could infer the past exposure to toluene. These biosensor cells did not degrade the target compound and, therefore, only detected the bioavailable fraction of toluene in the system above the threshold needed to trigger the response. Also in this study, the authors observed that the MBS was influenced by indigenous chemicals such as isoprene, which led to GFP induction.

## The bioavailability problem of very poorly water soluble compounds

The distinction between bioavailability and bioaccessibility becomes even more pronounced for polycyclic aromatic hydrocarbons (PAHs) than for BTEX. PAHs comprise a large group of compounds (>100 chemicals studied), most of which have no direct commercial use. They consist of two or more fused aromatic rings, have an elevated melting point and poor water solublity, and are typically formed during incomplete burning of organic material [48]. Combustion of coal, oil, gas and garbage are common sources of PAH production, but they can be found in cigarette smoke or grilled meat as well. PAHs in the environment mostly occur in sorbed form to organic matter or soil particles [48]. Apart from their acute toxicity, some PAHs are known or suspected carcinogens and they accumulate in animal tissue [35]. PAH biodegradation rates are strongly dependent on the chemical nature and number of aromatic rings, and are generally strongly limited by poor aqueous solubility [49]. For all these reasons, it is extremely important to have accurate measurements of PAH bioavailability and bioaccessibility, and in a variety of environments.

Bacterial MBS have mostly been designed for naphthalene(s) – a two-ring PAH of low molecular weight and moderate solubility in water – because of the known genetic details on naphthalene degradation [50-52]. Naphthalene-sensing MBS have typically applied the NahR regulatory protein in conjunction with the P*_sal_* or P*_nah_* promoters from the NAH7 plasmid of *P. putida* pPG7 [1,53]. Interestingly, use of this genetic circuit automatically leads to the detection of a metabolic ‘flux’ rather than of equilibrium concentration, since the chemical effector for NahR is not naphthalene but its metabolite salicylate [51]. Naphthalene needs to be metabolized by the MBS in order to generate internal salicylate, which then triggers reporter protein synthesis. Once in fully ‘activated’ state, the flux through the naphthalene pathway is high and internal salicylate concentrations will be low. Cells thus act as a sink for naphthalene and drive naphthalene diffusion toward them, a prerequisite for bioaccessibility assays [52]. A fluorene-targeting MBS was developed on the basis of randomly introducing a *luxAB* transposon into *Sphingomonas* sp. strain L-132 [54]. Although these cells could no longer completely metabolize fluorene as a consequence of the transposon insertion, they still partially transformed the compound and thus continue to act as sink. The strain detected fluorene concentrations as low as 200 μg/L (1.2 μM) in aqueous phase with a response time of between 30 min and 4 h. A phenanthrene-detecting MBS was constructed using *Burkholderia sartisoli* strain RP037. This strain produced GFP after contact with phenanthrene and naphthalene under control of the regulatory protein PhnR and its activated promoter P*_phnS_* [55]. PAHs have also been assessed with the help of a sensor-reporter strain induced by a toxicity-response invoked by PAHs [56,57].

MBS-assays for PAHs demonstrated that the cells are very sensitive to mass-transfer processes and are easily limited by the aqueous phase concentration. For example, the detection limit for naphthalene was lowered from 0.5 μM to 50 nM by using an MBS-assay in the gas-phase rather than in aqueous suspension [58,59]. This is due to the high volatility of naphthalene and the ∼10′000 times faster diffusion rates in air than in liquid [58]. Kohlmeier and colleagues then could further show that biosensor-reporter cells exposed to saturated naphthalene concentrations in aqueous solution without or with further naphthalene crystals produced the same maximum GFP reporter output after 4 hrs incubation time. However, cells in the assay with crystalline naphthalene continued to grow, leading to a dilution of the amount of GFP in the cells at incubations longer than 4 h as a consequence of the activated state of the naphthalene metabolic pathway (as explained above) [59]. This demonstrated that such cells can be used to differentiate naphthalene bioavailability (4 h measurement) and bioaccessibility (20 h measurement).

For PAHs with higher molecular weight, volatility is strongly reduced and the advantage for measuring with MBS in the gas phase is abolished. For this class of compounds the aqueous solubility strongly limits their bioavailability to the cells. Simple ‘calibration’ of the MBS-assay by incubating with different aqueous concentrations of the target compound no longer produces sufficiently different reporter activities in the cell. In that case, it becomes an option to calibrate the MBS on the basis of metabolic flux instead of equilibrium concentration (Fig. 3). We illustrated this possibility by using the *B*. *sartisoli* strain RP037 phenanthrene-sensing MBS [55]. *B*. *sartisoli* cells produce a stable GFP in response to phenanthrene metabolism. Probably because growth rates on phenanthrene are slower than the GFP synthesis rates, cells experiencing differences in phenanthrene flux produce more GFP over time. Four days-exposure times were required in order to obtain optimal signal-to-noise ratio, but this allowed us to calculate bioaccessible fractions for phenanthrene loadings in different materials, or from different surface areas [55].

**Box 1. Multi-target biosensor analysis**

Because a single bacterial host strain can be implemented with a wide diversity of genetic reporter circuits, multi-target arrays can be designed. The bacterium Escherichia coli is a long known laboratory ‘pet’ organism, whose growth and maintenance are easy and well controllable. For this reason, this bacterium has often been used as a host strain for sensor-reporter constructions and various reporter strains of E. coli are now available for a diversity of target chemicals. Since only small volumes of aqueous sample are required for an MBS-assay, a single sample can be tested against a battery of sensors with different target specificities. (a) Two liters of sea water were contaminated with 1% (v/v) of crude oil in a glass flask. Two hours after the addition of oil, water was sampled via the tap and analyzed for three compound classes in parallel, alkanes, BTEX and 2-hydroxybiphenyl. (b, c and d) Typical calibration curves with pure compounds in uncontaminated sea water. Output values obtained from the contaminated sample and from a spiked sample are indicated. Spiking consists of adding a known concentration of inducer (indicated by a star) that allows us to verify if the MBS is reporting satisfactorily. Data: R. Tecon and S. Beggah (unpublished).

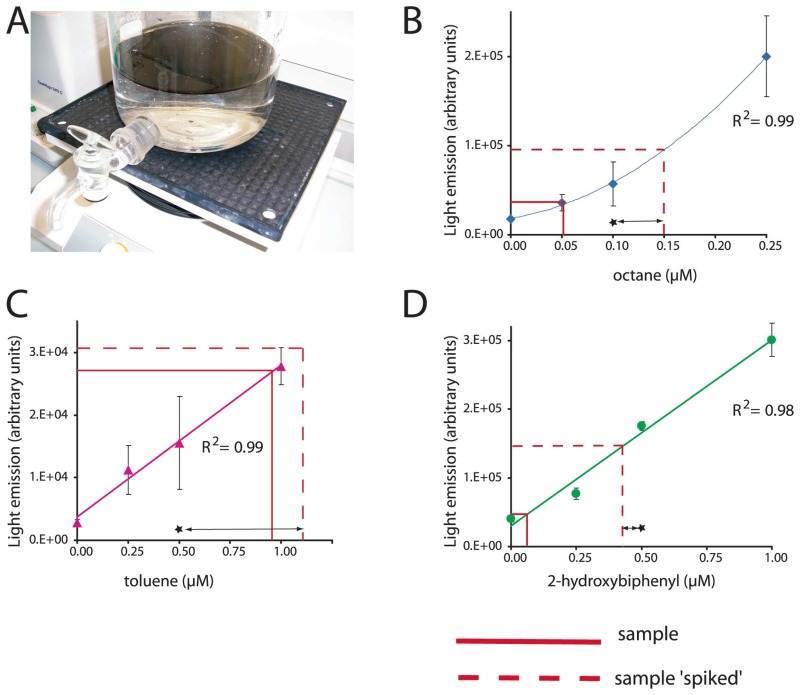

### MBS for toxic organic compounds

Phenol and derivatives are widespread contaminants whose sources are both natural and industrial. Phenol is massively produced and used as a starting material for synthetic polymers and fibers. Phenol is a strong irritant and long time exposure can cause a wide variety of health damages, including effects on the immune system [60]. Various phenol derivatives are known for their toxic action. Examples include 2-hydroxybiphenyl, a common disinfectant and fungicide, and 2,4-dichlorophenoxyacetic acid (2,4-D), a widely used herbicide that can cause nervous system damage in humans. One of the main metabolites of 2,4-D is 2,4-dichlorophenol (DCP), a proton shuttle and dissipator of membrane potential [61]. Various MBS have been developed to target phenolics, and have usually been based on bacteria degrading them.

Some of the earliest MBS for phenols were based on the regulatory protein DmpR and the P*_o_* promoter from the plasmid pVI150 of *Pseudomonas* sp. strain CF600. One MBS of this type, the strain *P*. *putida* KT2440::DmpR (pVI360), could be activated by phenol, cresols and some dimethylphenols, but did not respond to dichlorophenols or BTEX [62]. Similar MBS were constructed using the CapR-system from *P*. *putida* KCTC1453 [63] or the MopR-circuit from *Acinetobacter* sp. DF4 [64]. Modifying the sensor domain of DmpR by random mutations resulted in strains with an increased sensitivity to phenols and a broader range of detection [65].

Leedjarv *et al.* reconstructed an MBS based on the DmpR system (*P*. *fluorescens* OS8 [pDNdmpRlux]) and determined the bioavailable fractions of phenols in dump leachates and contaminated groundwater samples [66]. Since phenols are sufficiently water soluble, the MBS was calibrated in the classical ‘equilibrium’ mode (Fig. 3). The MBS-assay detected phenols in almost all samples, but the bioavailable fractions varied enormously, ranging from 0 to almost 100% of the total chemically-determined phenol amount in the sample. This demonstrated the great importance of taking compound bioavailability in samples into consideration for risk and bioremediation assesments. Sandhu and colleagues addressed the question of phenol bioavailability in the air nearby plant-leaves. Airborne phenol was detected using an MBS-assay directly on the plant leaves with *P*. *fluorescens* strain A506, expressing GFP under control of a mutated DmpR [65,67]. Their results showed that the sensors-reporter cells were able to detect phenol on plant leaves exposed to phenols in the vapour phase. Interestingly, the phenol concentration reported by the cells was more than tenfold higher than the chemically-determined phenol concentration in the air, which the authors interpreted as an accumulation of phenol on leaves.

Jaspers *et al.* developed an MBS-assay for the detection of 2-hydroxybiphenyl, a disinfectant and fungicide, based on the HbpR transcription activator of *Pseudomonas azelaica* [68]. Classical incubation assays in aqueous solution resulted in method detection limits of 0.5 μM, but this could be lowered some twentyfold by using a hypersensitive mutant of HbpR [69]. A hybrid assay was then developed which would detect bio-accumulation of 2-hydroxybiphenyl via crab urine, and this showed that the crabs concentrated 2-hydroxybiphenyl up to 100-fold after being exposed in contaminated seawater for one week (Lewis *et al*, unpublished).

Using a bacterium degrading 2,4-D and producing luciferase under control of the regulatory protein TfdR and P*_DII_* promoter from *Cupriviadus necator* JMP134 [61], Toba and Hay developed a solid-phase MBS-assay for the detection of 2,4-D in soil [70]. In this assay the sensor-reporter cells were spotted onto filter discs that were brought in direct contact for ≈ 60 min with the contaminated soil sample, after which the cells were retrieved and luciferase expression was analysed. Under appropriate moisture conditions, the MBS-assay detected 2,4-D at amounts between 1 and 50 mg/kg soil. Because these MBS cells degrade 2,4-D it would be conceivable to replace the luciferase reporter for GFP, expose for longer times and obtain a 2,4-D bioaccessibility assay – similar as outlined above for phenanthrene [55].

### MBS assays for polychlorinated biphenyls (PCBs) and oils

It is particularly challenging to obtain MBSs for PCBs, since no bacterial systems are known that can sense PCBs and trigger gene expression. PCBs are ubiquitous in the environment at low concentrations, are toxic and poorly degraded. PCBs have been shown to cause a large variety of health effects, which is more severe for the higher chlorinated congeners [71]. Because of the lack of appropriate sensory proteins in bacteria, most developments have relied on using co-induction involving further uncharacterized activator proteins. For example, a PCB-degrading *Ralstonia eutropha* served as a host strain for the construction of a MBS (*R*. *eutropha* ENV307 [pUTK60]). The strain expresses bacterial luciferase from the P*_bphA1_* promoter under control of an unidentified regulatory protein [72]. Although it is not clear whether this sensor-reporter bacterium directly senses chlorinated biphenyls or one of their metabolites, the MBS-assay enabled detection of biphenyl, monochlorinated biphenyls and Aroclor 1242 (a PCB mixture) in aqueous solution down to 1 mg/L. More recently, biosensor-reporter strains were used for PCB detection via its metabolites 3-chlorobenzoate [73] or chloromuconic acids [74]. Furthermore, the aforementioned HbpR system in *E*. *coli* was used in an assay to detect hydroxylated PCBs in aqueous solution and in human serum, with the idea of detecting metabolites in animals and human exposed to PCBs [75]. Interestingly, some hydroxylated PCBs were detectable at concentrations as low as 10 nM and serum as assay medium was found to result in higher reporter output in the assay [75]. Finally, most recently we ourselves showed that mutants of the HbpR regulatory protein can be obtained which enable direct detection of 2-chlorobiphenyl and triclosan [69]. None of those MBS-assays so far really addressed the issue of PCB bioavailablity or bioaccessibility, except indirectly the one using human serum [75].

Another compound class for which bioavailability and bioaccessibility are important issues, are alkanes. Alkanes are common constiuents of crude oil, natural gas and oil products, but come in a large variety of different chain lengths, branchings or cyclic forms (e.g., cyclohexane). Their environmental fate strongly depends on the number of carbon atoms, their solubility in water being inversely proportional to this number [35]. Although their acute and chronic toxicity are not extremely high, they form good indicators for oil pollution in the environment. Very few bacterial biosensor-reporter cells were constructed for alkane detection. The first described strain produced bacterial luciferase under control of the AlkS regulatory protein and P*_alkB_* promoter from *Pseudomonas oleovorans* [76]. Assays with the AlkS-MBS efficiently detected linear alkanes with chain lengths from C_6_ to C_10_ at nominal concentrations as low as 10 nM [76, 77]. Poor reporter signals were obtained with linear alkanes with longer chain lengths, with branched alkanes or cycloalkanes [76]. Because short-chain alkanes are very volatile, gas-phase based MBS-assays can be used like described for naphthalene detection. Consequently, decreasing the volume of gas phase in the assay helps to lower the apparent method detection limit with sensor-reporter cells in aqueous suspension [77]. An example of the functioning and calibration of this MBS is presented in Box 1. The detection of long-chain alkanes by MBS has proven to be very difficult, probably because of extremely low aqueous solubility (≈10 nM [78]), and thus very low bioavailability fraction. As a proof of principle, however, we previously studied the octane mass-transfer from a point source through the aqueous phase by using an *E*. *coli* strain with octane-inducible GFP formation [77]. This strain could not degrade but only detect octane and, therefore, could not form a sink driving further diffusion from the source. Octane diffusion gradients could be detected over a length of 2.5 cm in as short as 30 minutes [77].

### Conclusions

We illustrated here that microbial sensors, and in particular bacterial sensors, can easily be designed for a wide variety of purposes. For the sake of shortness, we have omitted any further examples of MBS for heavy metals or toxicity, which have been recently reviewed elsewhere [4,14]. Leaning on the tools of genetic engineering, today's huge genomic resources and the natural diversity within the microbial world, there is little limitation to our imagination for designing MBSs. In addition, we have shown that a plethora of assay forms can be easily conceived. Cultivation of bacterial cells – the heart of the MBS-assay - is easy, and production costs are very low. Method detection limits of MBS-assays, as we have demonstrated, are often in the nanomolar range, thereby competing effectively with existing chemical analytics. Despite these aspects, MBS-assays are still rarely applied outside research laboratories [79]. Convincing data have been produced which demonstrate field robustness, good measurement precision and accuracy of MBS-assays in comparison to chemical analytics, as in the case of arsenic in groundwater [80] or rice [81]. It is high time that regulatory authorities accept MBS as realistic alternative for a variety of analytical procedures, which would certainly help their implementation. In addition, MBS could offer excellent possibilities for assaying the complex nature of bioavailable and bioaccessible fractions in thousands of cases of severe and toxic pollution, which currently cannot be easily addressed. We are confident that MBS sensing-reporting technology will contribute to fill this gap in the near future.
